# Drug Eruptions: A Clinical Study of the Effect of Drugs upon the Skin

**Published:** 1887-06

**Authors:** 


DRUG ERUPTIONS: A CLINICAL STUDY OF THE EFFECT OF DRUGS UPON THE SKIN. *By* PRINCE A. MORROW, A.M., M.D., *Clinical Professor of Venereal Diseases, formerly Clinical Lecturer on Dermatology in the University of the City of New York, etc. New York:* WM. WOOD & Co. 1887. *Pp. 199.*

This is a little work of more value than many would suppose from its modest title. It gives in a convenient form the pathological results, so far as regards the skin, of both the ingestion and external application of medicamentous substances. The number of the latter is not only greater than most persons suppose who have given no attention to the subject, but the effects produced are so multiform and numerous that actually a large field in dermatology is covered by the subject. It is one naturally that chiefly concerns the dermatologist; but there is no man who aims to be an expert consultant in general medicine, or a careful diagnostician in any department of it, who can afford to ignore the important details here presented. As we look over the list of drugs capable of producing rashes, from arsenic and the potassium iodide that have been swallowed, to arnica and tar that have been externally applied, we can only look back with wonder as we speculate on the kind of diagnosis that our fathers in medicine would have made when confronted with the rashes that owe to these causes their sole existence.

Perhaps we might, with Tilden, take issue with our author in explaining, as he does, so many of these phenomena by supposing that they have a tropho-neurotic origin. We might claim for some of them that they had an intimate relation with vascular changes, and that some might be actually induced by the presence of toxic or irritative substances in the circulation. But we do not wish to be critical when such excellent results are spread before us.

We prefer, rather, to commend the work to the practitioner, not on the basis of so many others, as a book to be picked up and read during a spare half-hour. Not at all. It is a volume to be placed on the shelves for consultation; and in the face of an exceptional, singular or curious exanthem, it should be the first consulted.

